# Supplementary material for: Genomic analysis of Acinetobacter baumannii prophages reveals remarkable diversity and suggests profound impact on bacterial virulence and fitness
Source: Sci Rep. 2018 Oct 18;8:15346. doi: 10.1038/s41598-018-33800-5 (PMC6193963; doi:10.1038/s41598-018-33800-5)
Supplement: Supplementary file 1 — Supplementary information [file 41598_2018_33800_MOESM1_ESM.pdf]

Supplementary Information

Genomic analysis of *Acinetobacter baumannii* prophages reveals remarkable diversity and suggests profound impact on bacterial virulence and fitness

Ana Rita Costa, Rodrigo Monteiro, Joana Azeredo

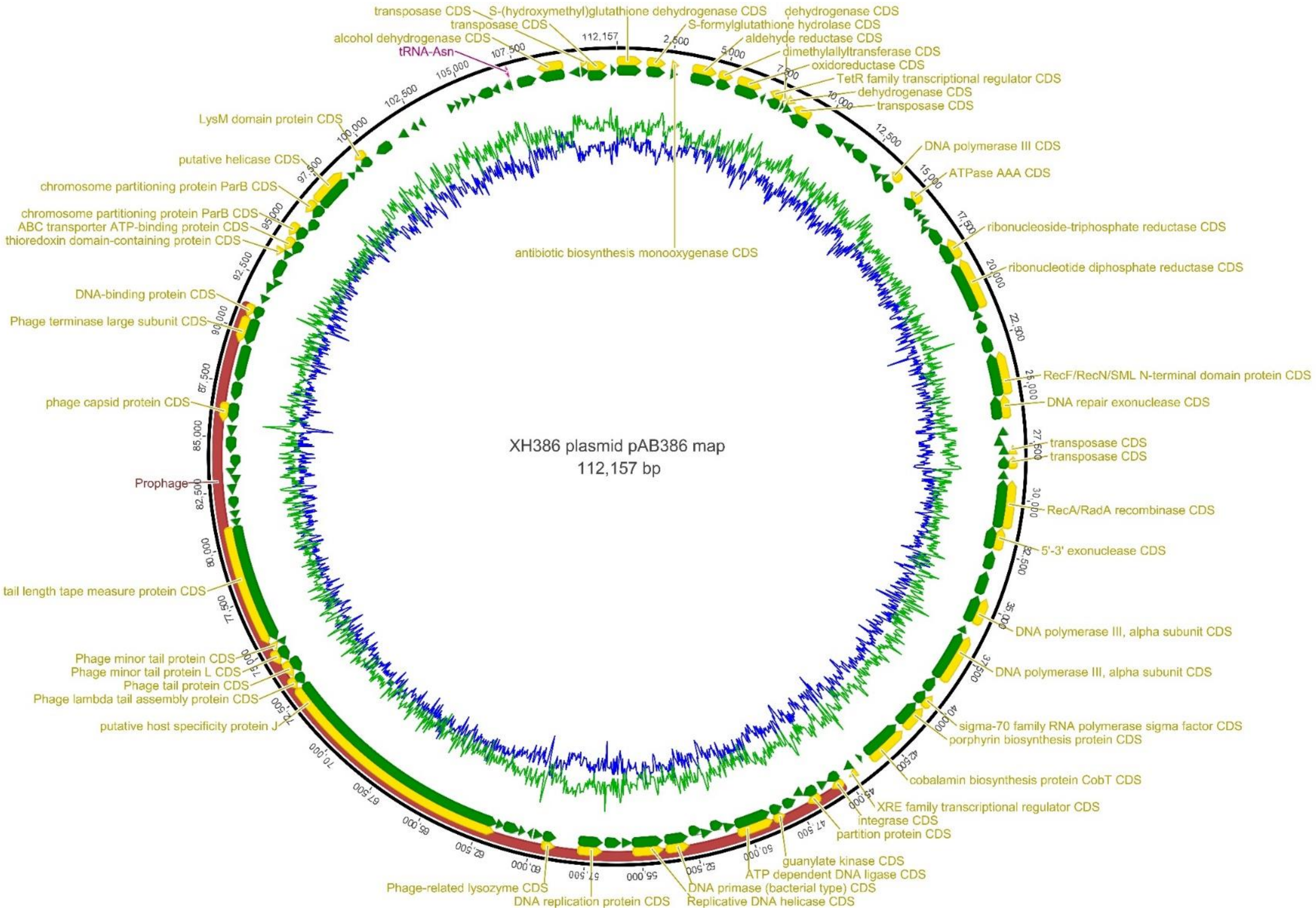

FIG S1. Map of plasmid pAB386 of *Acinetobacter baumannii* strain XH386. Green arrows: identified open reading frames; Yellow arrows: annotated coding DNA sequences (CDS); Pink arrow: tRNA; Red region: prophage sequence identified. Graphic for GC (blue) and AT (green) content in center.

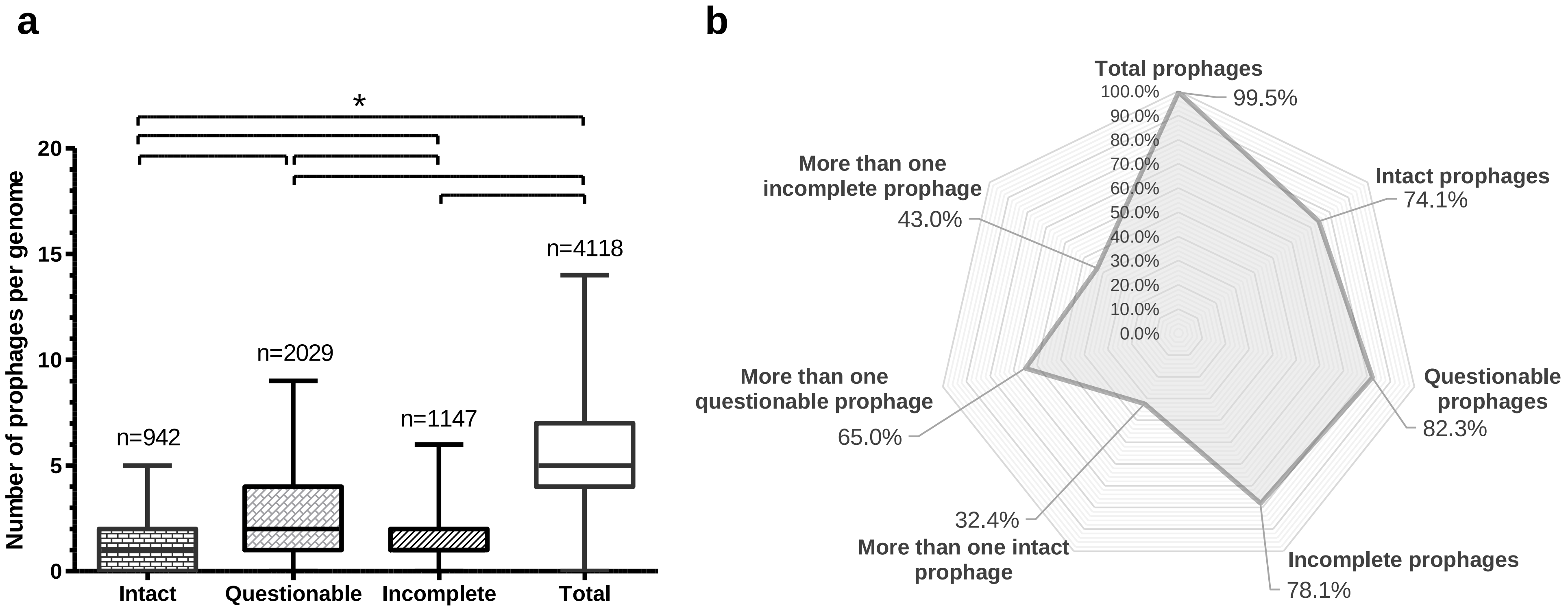

Figure S2. Prevalence of prophages in *Acinetobacter baumannii* genomes. (**a**) Whiskers plot of prophage frequency per bacterial genome. The horizontal line at the center of the whiskers plot represents the median. The bottom and top of the plot represent the first and third quartiles. The external edges of the whiskers represent the minimum and maximum number of prophages per genome. N is the number of prophages in the category. Significant differences (Tukey's test) of $P < 0.05$ are represented by *. (**b**) Prevalence of total prophages, "intact prophages", questionable prophages and incomplete prophages, more than one "intact prophage", more than one questionable prophage, and more than one incomplete prophage. Prevalence was determined considering a dataset of 795 *A. baumannii* genomes.

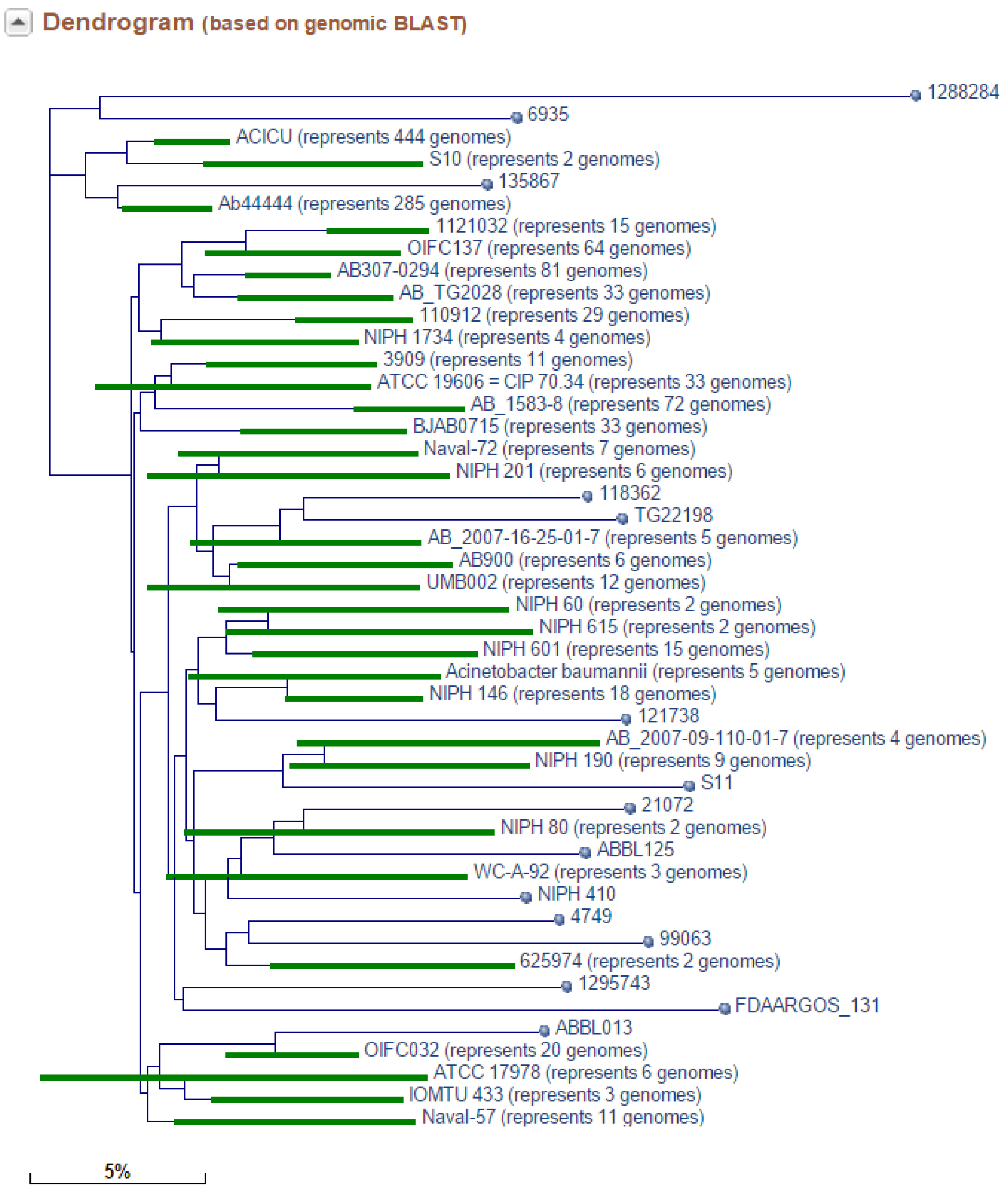

Figure S3. Dendogram of *Acinetobacter baumannii* retrieved from GenBank at the date of April 11, 2016. Representative strains from each branch were selected for detailed analysis of encoded prophages.
